# Experience in the management of sigmoid sinus thrombophlebitis secondary to middle ear cholesteatoma

**DOI:** 10.1186/s40463-023-00681-2

**Published:** 2023-12-19

**Authors:** Jing Fei, Xiao-Wen Peng, Ting-Yu Yang, Xue-Li Shen, Lin Gao, Na Liao, Lei-Ji Li

**Affiliations:** 1 Department of Otorhinolaryngology, Head and Neck Surgery, Affiliated Hospital of Southwest Medical University, 25 Taiping Street, Luzhou City, 646000 Sichuan Province China; 2 Department of Health Management Center, the Affiliated Hospital of Southwest Medical University, Luzhou, 646000 Sichuan Province China

**Keywords:** Sigmoid sinus thrombophlebitis, Cholesteatoma, Middle ear, Intracranial complications, Tympanoplasty

## Abstract

**Objective:**

To discuss the management of sigmoid sinus thrombophlebitis secondary to middle ear cholesteatoma.

**Methods:**

We retrospectively analyzed all cases of sigmoid sinus thrombophlebitis caused by middle ear cholesteatoma over a period of 7 years. 7 male and 2 female patients, ranging in age from 9 to 66 years, were diagnosed with sigmoid sinus thrombophlebitis by clinical presentation and radiological examination. By executing a modified mastoidectomy and tympanoplasty (canal wall-down tympanoplasty) to entirely remove the cholesteatoma-like mastoid epithelium, all patients were effectively treated surgically without opening the sigmoid sinus. All patients were treated with broad-spectrum antibiotics, but no anticoagulants were used.

**Results:**

9 patients had otogenic symptoms such as ear pus, tympanic membrane perforation, and hearing loss. In the initial stage of the surgery, modified mastoidectomy and tympanoplasty were performed on 8 of the 9 patients. 1 patient with a brain abscess underwent puncturing (drainage of the abscess) to relieve cranial pressure, and 4 months later, a modified mastoidectomy and tympanoplasty were carried out. Following surgery and medication, the clinical symptoms of every patient improved. After the follow-up of 6 months to 7 years, 3 patients were re-examined for MRV and showed partial sigmoid sinus recovery with recanalization. 4 months following middle ear surgery, the extent of a patient's brain abscess lesions was significantly reduced. 1 patient experienced facial paralysis after surgery and recovered in 3 months. None of the patients had a secondary illness, an infection, or an abscess in a distant organ.

**Conclusion:**

The key to a better prognosis is an adequate course of perioperative antibiotic medication coupled with surgical treatment. A stable sigmoid sinus thrombus can remain for a long time after middle ear lesions have been removed, and it is less likely to cause infection and abscesses in the distant organs. The restoration of middle ear ventilation is facilitated by tympanoplasty. It is important to work more closely with multidisciplinary teams such as neurology and neurosurgery when deciding whether to perform lateral sinusotomies to remove thrombus or whether to administer anticoagulation.

**Graphical abstract:**

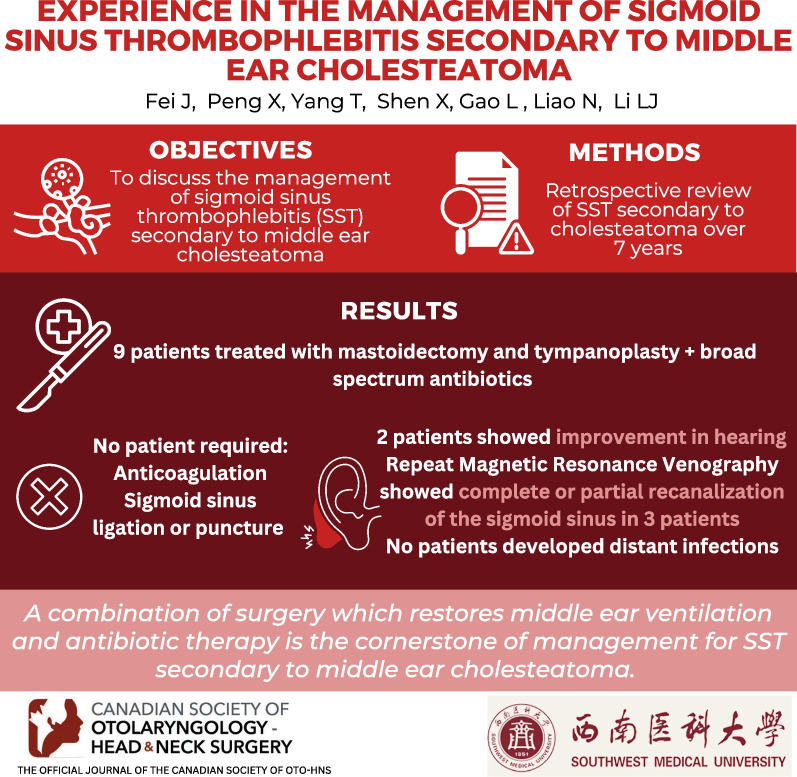

## Background

The mortality rate of sigmoid sinus thrombophlebitis is as high as 5%-10% [1–3]. Fever, headaches, edema of the optic papillae, and ear symptoms such as earaches, pus, hearing loss, and swelling or pain in the mastoid process are frequently observed in the early stages of the disease [4]. The uncontrolled use of antibiotics frequently masks these symptoms. There is still debate regarding the surgical treatments for otogenic sigmoid sinus thrombophlebitis, including sigmoid sinus exploration, incision of the sinus wall to remove the embolus, and sigmoid sinus ligation [1, 5]. By reviewing the clinical information of 9 patients with otogenic sigmoid sinus thrombophlebitis who were admitted to our hospital and combining it with relevant literature, we hope to assist readers in choosing an appropriate diagnosis and treatment strategy for this disease.

## Methods

The clinical data of all the patients diagnosed with otogenic sigmoid sinus thrombophlebitis in the Department of Otorhinolaryngology-Head and Neck Surgery of the Affiliated Hospital of Southwest Medical University between 2015 and 2022 were retrospectively analyzed. The prognosis of the patients was evaluated by follow-up imaging data and telephone interviews.

## Results

The 9 cases ranged in age from 9 to 66, with a duration of 2 months to 40 years. Of the 9, 7 were male and 2 were female. At the time of admission, there were 4 cases with febrile symptoms (Patients 2, 5, 7, 8), 5 cases with headache (Patients 2, 3, 5, 7, 8), 2 cases with vertigo (Patients 3, 8) and 3 cases with earache (Patients 2, 7, 9). All the patients had purulent ear leakage, including 3 with vomiting (Patients 2, 7, 8). During the course of the illness, there were 2 cases of impaired awareness (Patients 2 and 3), 1 case of optic papilla edema (Patient 3), and 1 case of diplopia (Patient 3) (Table 1).

**Table 1:** Clinical characteristics of patients with sigmoid sinus thrombophlebitis

	1	2	3	4	5	6	7	8	9
Sex	Female	Female	Female	male	Female	Female	Female	male	Female
Age	19	19	44	13	44	66	18	13	9
Site of symptoms	L	L	R	L	R	L	L	R	L
Fever	−	+	−	−	+	−	+	+	−
Headache	−	+	+	−	+	−	+	+	−
vertigo	−	−	+	−	−	−	−	+	−
Otalgia	−	+	−	−	−	−	+	−	+
Otorrhea	+	+	+	+	+	+	+	+	+
Vomiting	−	+	−	−	−	−	+	+	−
Disturbance of consciousness	−	+	+	−	−	−	−	−	−
Papilledema	−	−	+	−	−	−	−	−	−
Diplopia	−	−	+	−	−	−	−	−	−
Abducens nerve palsy	−	−	−	−	−	−	−	−	−

### Radiologic and laboratory findings

**Fig.1:**
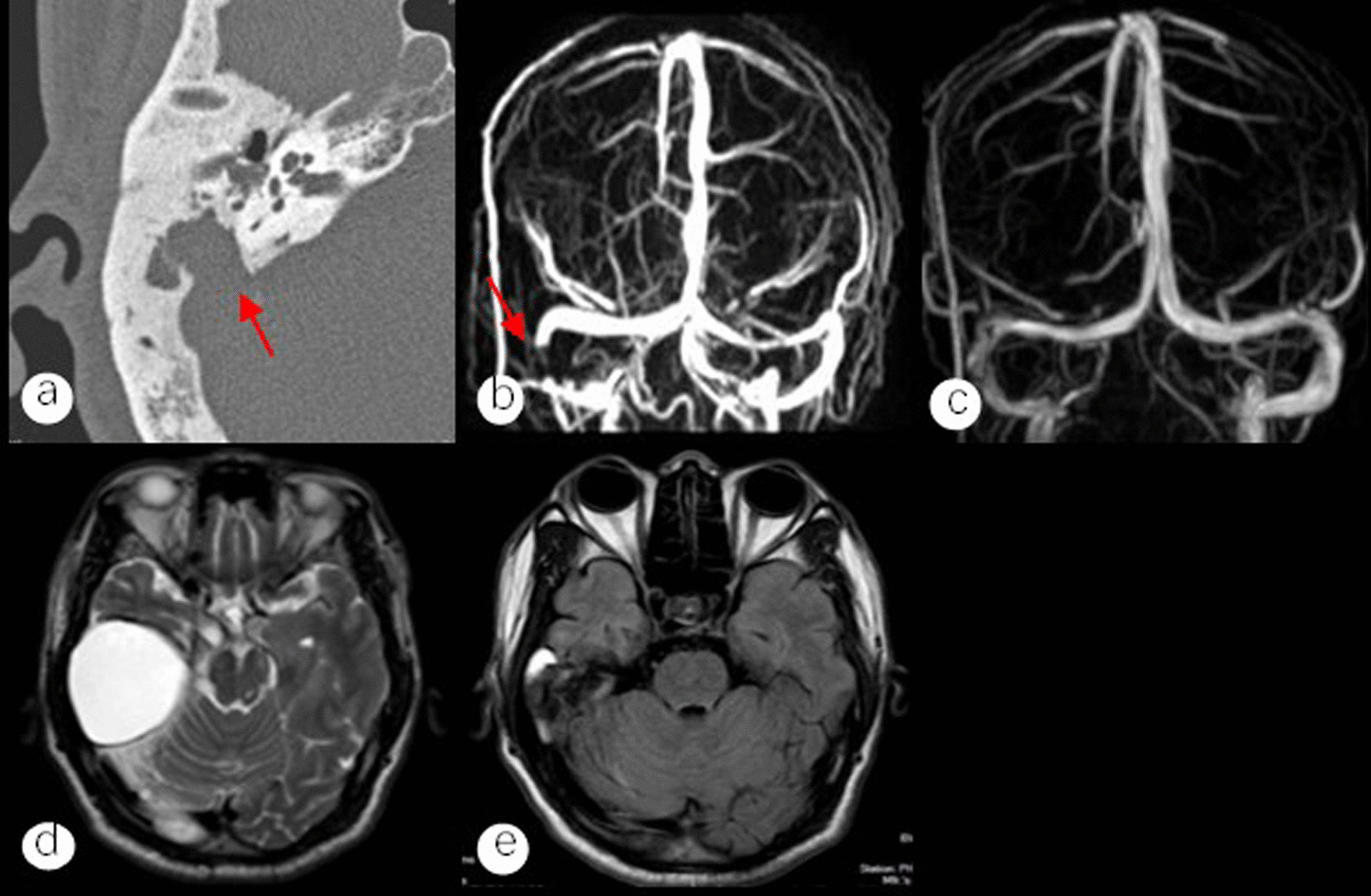
**a** Axial temporal bone CT with extensive bone destruction in the wall of the sigmoid sinus connected to the middle ear cavity (arrow); **b** Coronal MRV with no visualization of the right sigmoid sinus (arrow), confirming the diagnosis of sigmoid sinus thrombophlebitis; **c** MRV in sigmoid sinus thrombophlebitis patient 5 years after surgery suggests return of recanalization of right sigmoid sinus; **d** Axial MRI T1 image showing that this patient also had a combined temporal lobe brain abscess; **e** Axial MRI 3 months after tympanoplasty with significant resorption of the brain abscess lesion compared to the preoperative period

The temporal bone computed tomography (CT) in all patients showed middle ear abnormalities and varying degrees of bone destruction and defects between the bone wall of the sigmoid sinus and the mastoid cavity (Fig. 1a). Except for patient 2, whose enhanced **magnetic resonance imaging (MRI)** suggested the possibility of thrombus formation in the sigmoid and transverse sinuses, none of the patients' cranial MRIs revealed any apparent evidence of thrombus. The extent of the temporal lobe brain abscess was visible on patient 3's MRI (Fig. 1d). Before surgery, 8 patients received MRV examinations (Fig. 1b). Of these, 3 cases involved only the sigmoid sinus, 4 cases involved both the sigmoid and transverse sinuses, and 1 case involved the transverse, sigmoid, and superior sagittal sinuses (Table 2).

**Table 2:** Radiologic findings of patients with sigmoid sinus thrombophlebitis

		1	2	3	4	5	6	7	8	9
CT	Pre–operative	COME, SS anterior wall bone and mass destruction	COME, SS、TSA small amount of air shadow in the walking area	COME, Temporal lobe brain abscess with bone destruction in the anterior wall of the SS	COME, SS bone destruction of the anterior wall and abscess formation behind the ear	COME	COME, SS anterior wall bone and mass destruction	COME, SS anterior wall bone and mass destruction and intracranial involvement of the lesion	COME, SS anterior wall bone destruction	COME, SS anterior wall bone destruction
	Postoperative	–	Post–operative manifestations of otitis media, resumption of air in the middle tympanic chamber, and eustachian tube	Reduction in the extent of temporal lesions	–	–		–	Post–operative manifestations of otitis media, resumption of air in the middle tympanic chamber, and eustachian tube	–
MRI	Pre–operative	Middle ear mastoiditis	Enhanced MRI suggests possible SS and TS thrombosis	Temporal lobe brain abscess	Cholesteatoma mastoiditis of the middle ear	Cholesteatoma mastoiditis of the middle ear	Cholesteatoma mastoiditis of the middle ear	Middle ear cholesteatoma with lesion breaking through the wall of the sigmoid sinus	Otitis media, meningitis	–
	Postoperative	–	Small piece of abnormal signal in SS walking area	Decreased volume of temporal lobe brain abscess	–	–		–	Post–operative manifestations of otitis media	–
MRV	Pre–operative	TS slim, SS occluded	SS, TS occlusion	SS occlusion	TS slim, SS, SGS occlusion	SS slim	SS slim, TS occluded	TS slim, SS occluded	–	SS occlusion
	Postoperative	–	SS, TS section re–pass	SS Partial re–pass	–	–		–	SS Re–pass	–

*COME* cholesteatoma of the middle ear, *IJV* internal jugular vein, *SGS* sagittal sinus, *SS* sigmoid sinus, *TS* transverse sinus

Among the 9 patients, 5 had elevated leukocytes, 3 had anemia, and none had thrombocytosis. Bacterial cultures were taken from the external auditory canal secretions, and there were 3 positive results for Enterococcus cotton candy, Serratia marcescens, and Staph epidermidis. Blood cultures were positive in 2 cases. In one case, Enterococcus faecalis and Prevotella regia were both cultured and in another, only Enterococcus cotton candy was cultured. Streptococcus chimera was detected in the retroauricular subperiosteal abscess puncture fluid culture of 1 case (Table 3). A postoperative lumbar puncture was performed on 1 patient (Patient 2) who had normal intracranial pressure, and no obvious abnormalities were found in the cerebrospinal fluid biochemistry or culture.

**Table 3:** Treatment of patients with sigmoid sinus thrombophlebitis

	1	2	3	4	5	6	7	8	9
Bacteriological culture	–	Enterococcus faecalis, Prevotella regia (blood culture)	Staphylococcus epidermidis (brain abscess puncture fluid), Serratia marcescens (ear secretion)	Aspergillus chimera (abscess pus behind the ear)	Enterococcus faecalis (ear secretion, blood culture)	–	–	–	Staphylococcus epidermidis (ear secretion)
Antibiotics/Day	CXM/8 CPZ/7	CXM/3 CRO/22 CPZ/14	CXM/21 CPZ /7	CXM/9 CRO + ORN/5 CPZ/7	PIS + MFX /14 CPZ/7	CXM/8 CPZ/7	LOFX /1 CAZ + ORN /7 CPZ/7	CAZ/13 CPZ/7	CAZ/10 CPZ /7
Surgery	M + T + L	M + T	D, M + T	M + T + L	M + T	M + T	M + T + L	M + T	M + T + L

*CXM* Cefuroxime, CPZ Cefprozil, *CRO* Ceftriaxone, *CAZ* Ceftazidime, *ORN* Ornidazole, *PIS* Piperacillin sulbactam, *MFX* Moxifloxacin, *LOFX* Levofloxacin, *M* modified radical mastoidectomy, *T* canal wall-down tympanoplasty, *L* labyrinth fistula repair, *B* brain abscess puncture and drainage

### Treatment

Anti-infective medication was administered to all the patients. The usage of antibiotics lasted an average of 23 days (15–39 days). Tertiary cephalosporins were most frequently utilized, sometimes in combination with ornidazole in serious infections. All medications were given intravenously while the patient was hospitalized. The patients were often instructed to take it orally for at least 7 to 14 days after being discharged (Table 3).

All 9 patients experienced 1 to 2 temporal bone complications, including 2 cases of retroauricular subperiosteal abscess, 1 case of peripheral facial paralysis (House-Brackmann grade II) following surgery, which improved to grade I after three months, and 4 cases of labyrinthine fistulae. Of the 4 cases, the horizontal semicircular canal, the superior semicircular canal, and the posterior semicircular canal were all invaded in 1 case, while only the horizontal semicircular canal was involved in 2 cases, and the posterior semicircular canal was involved in 1 case. There was 1 patient who had a brain abscess in combination. Due to its obvious intracranial occupying effect, external drainage of brain abscess by puncture was performed in neurosurgery at stage I. The patient's symptoms, such as headache, nausea, and vomiting, improved after the operation. A modified mastoidectomy and type III tympanoplasty were carried out after a follow-up of MRI 4 months later, revealing a substantially reduced but still present brain abscess. The remaining 8 patients had tympanoplasty, auditory chain repair, and modified mastoidectomy in stage I(Table 3). No sigmoid sinus puncture, sigmoid sinus ligation, embolization incision, or anticoagulation during the perioperative period was performed on any of the patients.

**Fig. 2:**
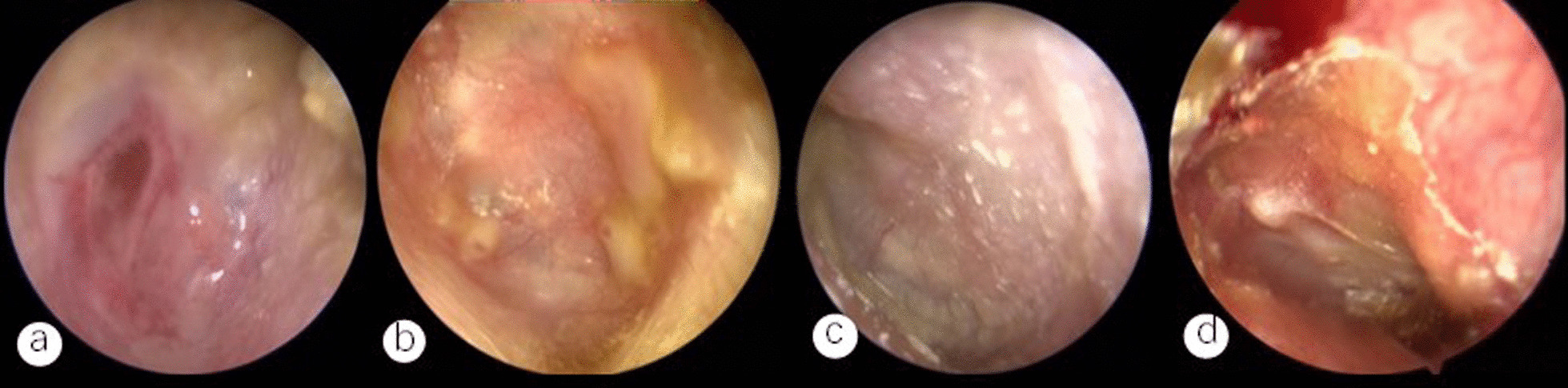
**a** Perforation of the tympanic membrane; **b** Three months after the surgery, the tympanic membrane was intact; **c** The preoperative tympanic membrane showed unclear marks; **d** 3 months after the surgical repair of the tympanic membrane, the marks of the tympanic membrane became clear

**Fig. 3:**
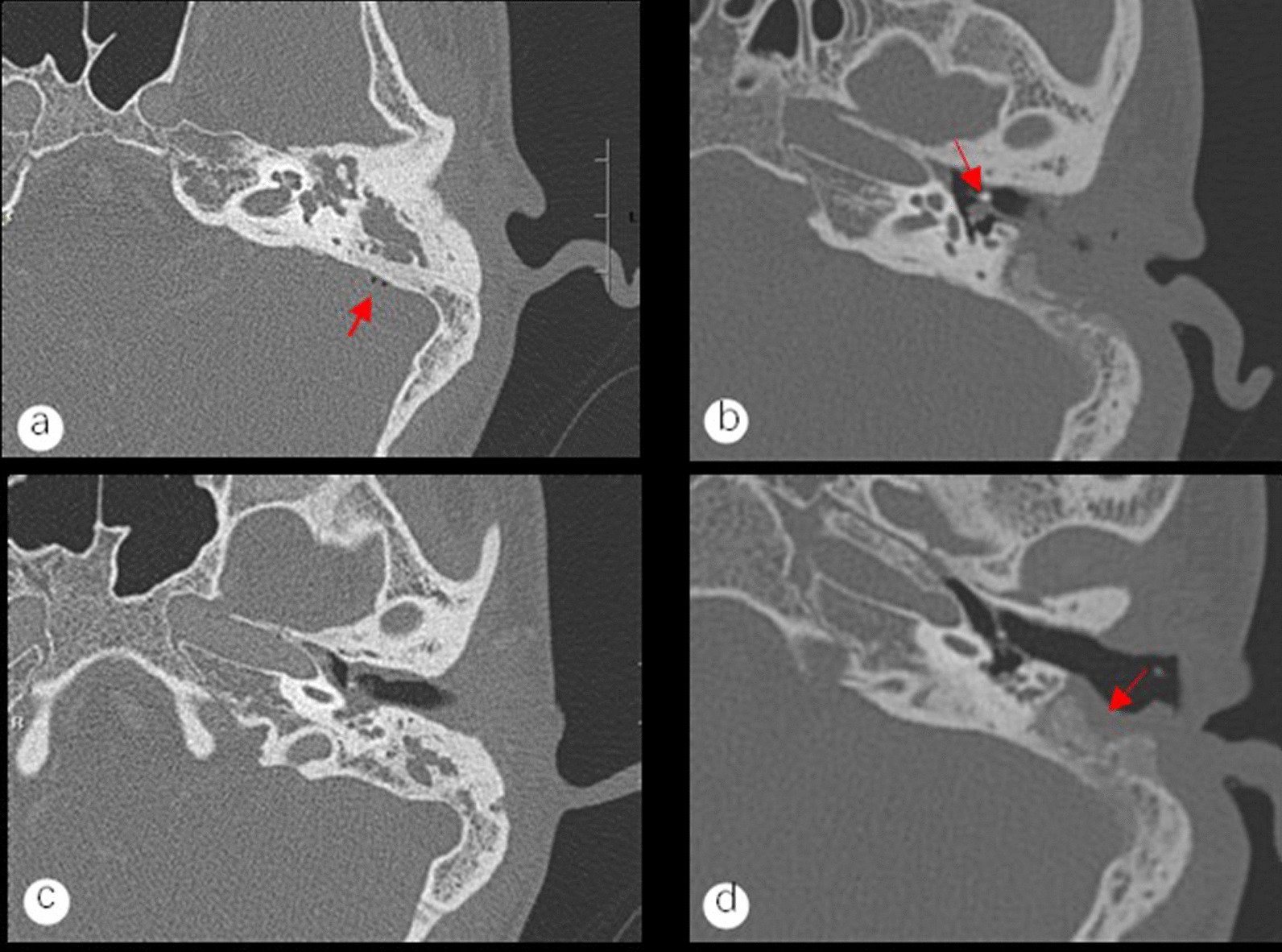
**a** The patient's preoperative axial temporal bone CT shows bone destruction of the long pedicle of the anvil, with the air shadow around the sigmoid sinus shown by the arrow. **b** The patient's temporal bone CT was repeated 3 months after tympanoplasty. The reconstructed cartilage piece of the auricular cavity shown by the arrow established contact with the vestibular window and isolated in the middle tympanic cavity. **c** The patient's preoperative axial temporal bone CT with soft tissue filling of the eustachian tube bullae. **d** The middle ear cavity was restored to contain air 3 months after surgery, and the reconstructed external auditory canal was in good shape. The arrow shows the grafted fascia and the filled bone powder, and the fascia has survived

In 1 of the 4 patients with fever, the temperature returned to normal after switching from ceftizoxime to ceftriaxone based on the drug sensitivity findings before surgery, and in the other 3 patients, the temperature did so within a day after the operation. All the patients who underwent surgery had satisfactory mastoid cavity epithelialization, and the transplanted fascia lasted after six months to seven years of follow-up (Fig. 2). A postoperative CT revealed that the middle ear cavity had restored its ventilatory function and that there had been no cholesteatoma recurrence (Fig. 3b, d). 7 patients showed no discernible change from the preoperative period, whereas 2 patients demonstrated improvement in hearing compared to the preoperative period. Repeat MRV indicated that the sigmoid sinus resumed recanalization in 1 patient, partial recanalization in 2 patients (Fig. 1c), and stable thrombus formation in the 6 patients who showed no significant change from the preoperative control. None of the 9 patients developed an infection or an abscess that was distant from their organs.

## Discussion

The infective symptoms of sigmoid sinus thrombophlebitis described in the current literature may not be typical [6]. In our cases, all the individuals exhibited ear leaks, however, only 5 of 9 also had headaches or fevers. There were 2 patients (Patients 2 and 3) with impaired consciousness, in contrast to the symptoms noted by Mete Iseri, Ireneusz, et al. [7–9]. All of our patients were primarily defined by ear symptoms, with minimal cranial nerve involvement. Optic papillary edema was only present in 1 patient with a combined brain abscess, which may be connected to the early use of antibiotics.

Various studies have supported the link between sigmoid sinus thrombophlebitis and various temporal bone complications [10]. All the patients had 1 to 2 temporal bone complications, and meningitis was the most frequent intracranial comorbidity. In contrast to individuals with combined brain abscesses, this group of patients frequently displayed varying fever and headache. The signs and symptoms of temporal bone complications might sometimes be subtle in an era where antibiotic use is common. Therefore, when treating patients with sigmoid sinus thrombophlebitis, otolaryngology experts must be vigilant in identifying other temporal bone problems.

For sigmoid sinus thrombophlebitis, the standard imaging method is the temporal bone CT. It displays the extent of the primary lesion in the middle ear as well as the level of bone erosion in the sigmoid sinus' anterior wall [10]. The anomalies of MRI's presentation have extremely high tissue resolution and sensitivity to blood flow and depend on when thrombosis occurs. T1-weighted images exhibit significant signals in the subacute phase (1–2 weeks) [11]. However, due to the progressive mechanization of the thrombus in patients with a duration of longer than two weeks, MRI frequently fails to demonstrate thrombosis. Only Patient 2's increased MRI raised the likelihood of sigmoid sinus and transverse sinus thrombosis in this group; the preoperative MRI results for the other patients did not raise any such concerns. Patient 5 is one example of a diagnosis that would have been missed if we simply used temporal bone CT and cranial MRI. The anterior wall of the sigmoid sinus did not appear to have any clear areas of bone degradation on the temporal bone CT scan, and neither did the scan on the MRI. However, the patient's preoperative MRV indicated that the sigmoid sinus was thin, and during surgery, the front wall of the sigmoid sinus had a bone defect of approximately 1 × 2 cm^2^. Additionally, this patient experienced typical sigmoid sinus thrombophlebitis symptoms such as headache, hyperthermia, and sepsis. Compared with conventional MRI, MRV can confirm cerebral venous sinus thrombosis and clarify the extent of thrombus, site of occurrence, and degree of stenosis. Additionally, recanalization of blood arteries following therapy can be seen by MRV, which can increase the accuracy of the diagnosis [1, 10, 11]. It is a crucial adjunct technique for detecting cerebral venous sinus thrombosis.

It is unclear how long sigmoid sinus thrombophlebitis patients will need to take antibiotics. Our cases had an average duration of antibiotic usage of 27 days, which included at least 1 week of home antibiotics, which is largely in line with the literature [12]. Due to the widespread usage of antibiotics, the bacterial culture positivity was only moderate. Although we grew 3 g-positive and 3 g-negative bacteria, most researchers [13] found that gram-negative bacteria were more prevalent. 2 of the 9 cases had mixed flora infections, which are frequently present in patients with sigmoid sinus thrombophlebitis. As a result, we frequently choose antibiotics that have a broad spectrum and are simple to cross the blood–brain barrier. The bacterial culture sensitivity test and clinical symptoms should be taken into account when determining the extent of antibiotic treatment.

Surgery is frequently the mainstay of treatment for otogenic sigmoid sinus thrombophlebitis. Depending on whether the sigmoid sinus is incised for embolization, it is classified as conservative or radical [14]. According to certain research, radical surgical operations such as jugular vein ligation or phlebotomy for thrombolysis should only be undertaken if infectious thromboembolic features are unmistakably progressing [15, 16]. Internal jugular vein ligation and incisions for thrombolysis were both shown to be ineffective in the treatment of individuals with sigmoid sinus thrombophlebitis, according to Thorsten et al.'s retrospective analysis of 6 patients with sigmoid sinus thrombophlebitis [17]. In our group, only otologic surgery was conducted on all 9 patients because there were no clear progressing indications of infective thrombus (Figs. 2 and 3). All 9 patients had a good prognosis and positive postoperative results. None of them showed infection or thrombus dislodging away from the organ. Therefore, we advise against performing invasive procedures such as lateral sinusotomy for thrombus removal or ligation in cases when there are no overt signs of an infective thrombus.

**Fig.4:**
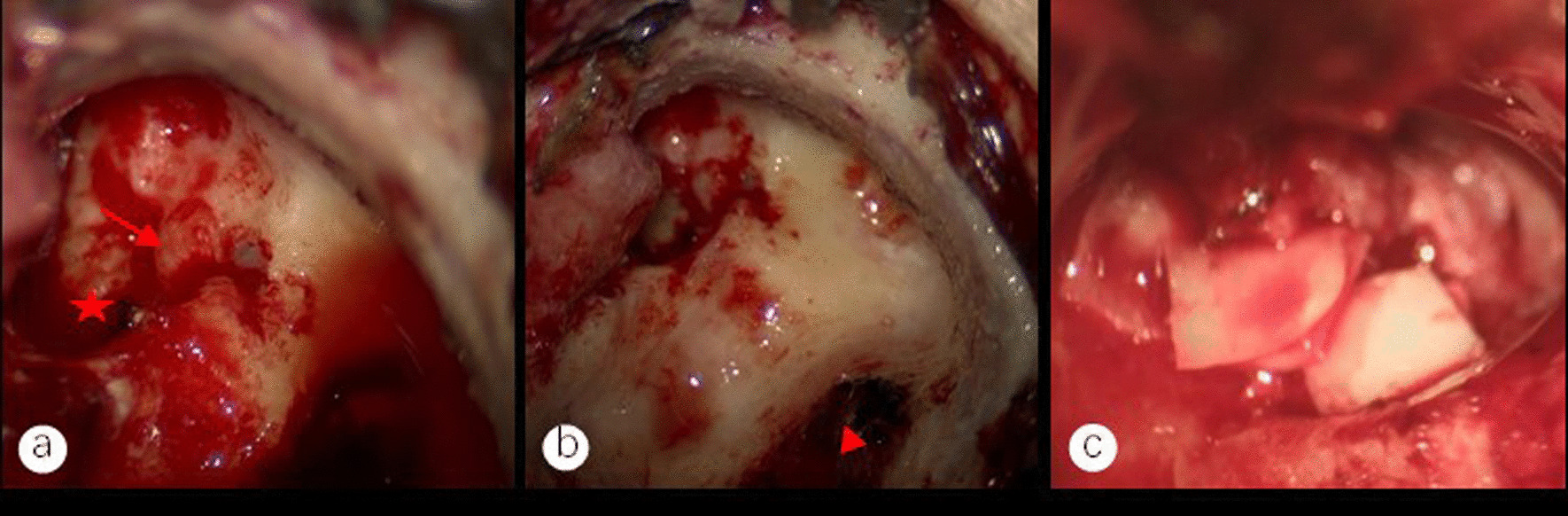
**a** The red arrow indicates the exposed facial nerve canal and the red star indicates the stapedial base. The granulation or cholesteatoma epithelium on the surface of the exposed facial nerve is removed without damaging the facial nerve sheath, and the granulation tissue that cannot be removed from the stapes base is cauterized with 10W bipolar cautery to prevent recurrence of cholesteatoma. **b** The red triangle indicates the exposed sigmoid sinus vein wall, and the cholesteatoma epithelium on the vein wall is removed, preserving the cholesteatoma basilar membrane. **c** The auditory chain is reconstructed through the cartilage piece of the ear cavity and a connection to the vestibular window is established

We think that modified mastoidectomy and tympanoplasty in stage I should be carried out as soon as possible in cases with sigmoid sinus thrombophlebitis secondary to middle ear cholesteatoma. The Trautmann method enables removal of the abnormal tissues from the middle ear and appropriate exposure of the surgical cavity. When the cholesteatoma epithelium and inflammatory granulation tissues were present in the damaged vein wall, only the outer membrane of the cholesteatoma was also removed. Using cartilage from the auricular nail cavity, type III tympanoplasty was performed in every patient in our group in stage I (Fig. 4c). Tympanoplasty staging is a subject of debate. When there are serious mucosal lesions, according to James et al. [18], tympanoplasty staging is necessary. According to Luca et al. [19], tympanoplasty in stage II has a recurrence incidence of up to 70%, however, opting for tympanoplasty in stage I nearly eliminates recurrence and lowers the need for hospitalizations and procedures. This approach is affordable and successful. Therefore, we think that tympanoplasty and modified mastoidectomy should be carried out as soon as possible. The key to treating this condition, particularly the sick tissue surrounding the eustachian tube aperture, is to completely remove the middle ear infective lesions. This is crucial for enhancing the hearing prognosis and aiding in restoring the middle ear's ability to hold air following tympanoplasty [20].

The role of anticoagulation therapy for sigmoid sinus thrombophlebitis is unclear. Six sigmoid sinus thrombophlebitis patients were included in the analysis by N. de Oliveira Penid et al. [21], 3 of whom received anticoagulation therapy and 3 of whom did not. 1 patient who was not taking anticoagulants and underwent follow-up testing experienced sigmoid sinus recanalization, indicating that the use of anticoagulants is not a factor in this condition. Anticoagulants have the risk of causing thrombocytopenia, worsening operating cavity bleeding, and encouraging the production of septic emboli. We did not administer anticoagulation, which is in keeping with many recommendations in the literature, because there were no evident signs of thrombosis in our case, the infection was swiftly under control following middle ear surgery, and the temperatures all quickly returned to normal range. In our opinion, anticoagulation should be performed only in those with persistent fever despite appropriate surgical intervention, extensive thrombus involvement or cerebral venous infarction, pulmonary embolism, or persistent sepsis [22], and the dosage and intensity of anticoagulation should be guided by a neurologist.

## Conclusion

In conclusion, we think that a combination of the patient's medical history, neurologic and otogenic symptoms, signs, and radiographic imaging should be used to make the diagnosis of sigmoid sinus thrombophlebitis secondary to middle ear cholesteatoma. MRV is the most important and intuitive means of recognizing and diagnosing sigmoid sinus thrombophlebitis and adopting the appropriate treatment plan. Based on drug sensitivity tests, enough antibiotics that may cross the blood–brain barrier are employed to treat sigmoid sinus thrombophlebitis secondary to middle ear cholesteatoma. An adequate quantity of blood–brain barrier-crossing antibiotics should be chosen for the treatment of sigmoid sinus thrombophlebitis based on the results of the drug sensitivity test. Along with effective anti-infection measures, a mastoidectomy should be carried out as soon as possible. After the middle ear lesions have been completely removed, tympanoplasty can be performed in stage I, which helps restore the middle ear's ventilation function. When deciding whether to perform lateral sinus incision and embolization ligation and whether to employ anticoagulant therapy, collaboration with interdisciplinary teams such as neurology should be reinforced.

## Data Availability

The datasets generated and analyzed during the current study are not publicly available but are available from the corresponding author at reasonable request.
